# Household mental distress and child cognitive development in Ghana

**DOI:** 10.3389/fpsyg.2026.1840678

**Published:** 2026-07-29

**Authors:** Elizabeth Asiedu, Ami Adjoh-Baliki, Alexander Opoku

**Affiliations:** 1 Howard University, Washington, DC, United States; 2 American Institutes for Research, Arlington, VA, United States; 3 Lancaster University, Accra, Ghana

**Keywords:** child cognitive development, Ghana, household mental distress, human capital, instrumental variables, psychological wellbeing

## Abstract

**Introduction:**

Household mental wellbeing plays an important role in children’s cognitive development, yet evidence from developing countries remains limited. This study examines the relationship between household mental distress and children’s cognitive development in Ghana.

**Methods:**

We use nationally representative longitudinal data from the Ghana Socioeconomic Panel Survey (2009-2010, 2013-2014, and 2018), comprising 8,755 children aged 5–17 years. Children’s cognitive ability is measured using a composite index derived from the Digit Span Forward, Digit Span Backward, and Raven’s Progressive Matrices assessments. Household mental distress is measured using the average Kessler Psychological Distress Scale (K10) score of adults in the household. To address potential endogeneity, we employ a leave-one-out community mental distress instrumental variable and supplement the analysis with alternative instrumental-variable specifications, treatment matching, and placebo tests.

**Results:**

Across all estimation approaches, children living in mentally distressed households exhibit significantly lower cognitive performance than children in non-distressed households. Estimated effects range from 0.43 to 0.49 standard deviations and are observed among both pre-teens (5-12 years) and adolescents (13-17 years). Analyses using a continuous measure of household mental distress produce similar findings consistent with a dose-response relationship.

**Discussion:**

Household mental distress is strongly associated with children’s cognitive development in Ghana. Policies aimed at improving children’s developmental outcomes may therefore benefit from greater attention to adult mental health within households.

## 1 Introduction

Cognitive, social, and emotional development during childhood and adolescence lays the groundwork for building human capital and long-term economic productivity (Cunha and Heckman, 2007; Heckman, 2006). Skills such as memory, executive functioning, and fluid reasoning significantly affect educational achievement, skill acquisition, and success in the labor market (Diamond, 2013; Duncan et al., 2007). In line with this, the United Nations Sustainable Development Goal 4 aims to ensure that all children develop the cognitive foundations necessary for effective learning and active participation in society. However, many children, especially in developing countries, continue to encounter socioeconomic and psychosocial challenges that limit their cognitive potential.

There is ample empirical evidence that the mental health of parents and caregivers is closely linked to children’s developmental outcomes (Bennett et al., 2016; Leijdesdorff et al., 2017; Wolicki et al., 2021). Psychological distress in adults can negatively impact caregiver-child interactions, increase family conflict, weaken emotional support networks, and diminish cognitive stimulation in the home environment (Huhtala et al., 2014; Howard et al., 2023). Furthermore, research in developmental neuroscience indicates that prolonged exposure to stressful family environments can affect neural systems related to memory, executive functioning, and emotional regulation (Luby et al., 2013; Xerxa et al., 2021). Together, these findings suggest that the psychological wellbeing of adults in children’s environments plays a crucial role in shaping cognitive development.

However, several important gaps remain in the literature. Most existing studies primarily focus on the mental health of individual caregivers—particularly mothers—rather than on the broader psychological environment surrounding children (Mensah and Kiernan, 2010). Such analysis may not adequately reflect caregiving arrangements in many developing-country settings, where child-rearing responsibilities are often shared among parents, grandparents, aunts, uncles, and other household members (Annim et al., 2015). Furthermore, most of the evidence comes from high-income countries, raising concerns that the results may not be applicable to developing countries. Another relevant issue is that empirically establishing the relationship between household psychological distress and children’s cognitive development can be challenging because observed differences in cognitive performance may be attributable to household psychological distress itself rather than to related family circumstances. Finally, although cognitive development has frequently been examined during early childhood, environmental stressors continue to affect cognitive functioning throughout later childhood and adolescence (Blakemore, 2019; Tooley et al., 2021).

These gaps are especially relevant in Ghana and similar developing countries, where mental health conditions are prevalent, access to treatment is limited, and households often serve as the primary source of care and support (World Health Organization [WHO], 2022). Children in these environments are likely to be influenced not only by their parents’ psychological wellbeing but by the emotional functioning of other adults in the household who play a care-giving role. Focusing solely on individual caregivers may lead to an incomplete understanding of the psychosocial environments that influence children’s development. Therefore, this study takes a household-level perspective, conceptualizing mental distress as a shared psychological climate that can influence children’s developmental experiences. By shifting from individual caregivers to the broader household environment, the study enhances our understanding of how family psychological conditions impact children’s cognitive development in developing countries. Furthermore, by employing analytical methods designed to minimize the influence of confounding factors, the study contributes to ongoing efforts to better understand the relationship between household mental distress and children’s cognitive development.

This study examines the relationship between household mental distress and children’s cognitive development in Ghana. Specifically, we examine whether children living in psychologically distressed households exhibit lower cognitive performance. Additionally, we investigate whether this relationship holds for two development stages: pre-teen (ages 5–12) and teen (ages 13–17).

## 2 Data and variables

The study uses data from the Ghana Socioeconomic Panel Survey (GSPS), a nationally representative longitudinal household survey conducted in three periods: 2009–2010, 2013–2014, and 2018. The GSPS tracks households over time, collecting detailed information on demographics, education, health, and socioeconomic conditions. This survey is designed to monitor changes in these socioeconomic conditions and the overall wellbeing of Ghanaian households. The GSPS has been used in several studies to analyze mental health issues in Ghana (e.g., Owoo and Lambon-Quayefio, 2021; Vitellozzi et al., 2024; Amenah et al., 2025).

### 2.1 Dependent variable

The dependent variable is a composite measure of children’s cognitive ability constructed from three standardized cognitive assessments administered in all three waves of the GSPS: the Digit Span Forward test (DSF), the Digit Span Backward test (DSB), and Raven’s Pattern Cognitive Assessment (RPCA). The DSF measures short-term and working memory capacity, while the DSB focuses on working memory and executive functioning. Both digit-span tests assess children’s recall abilities. The RPCA measures non-verbal reasoning and pattern recognition in a manner that is designed to be culturally neutral.^1^ Together, these assessments capture complementary dimensions of cognitive functioning that are central to children’s cognitive development.

The assessments were administered across survey waves using standardized protocols. To construct a composite measure of cognitive ability, each test score was first standardized using the pooled sample from all three survey waves, ensuring that scores were expressed on a common metric across time. Following previous studies that combine multiple cognitive assessments into a single measure of cognitive functioning (von Grafenstein et al., 2023; Kramer et al., 2021), principal component analysis (PCA) was applied to the standardized DSF, DSB, and RPCA scores. The first principal component had an eigenvalue of 2.13 and explained approximately 71% of the total variance across the three assessments. Factor loadings were positive and of similar magnitude for DSF (0.59), DSB (0.60), and RPCA (0.54), indicating that the component reflects a common underlying dimension of cognitive ability rather than test-specific variation. Consistent with standard practice, the first principal component was retained as the composite cognitive index. Additional psychometric analyses further support the use of a composite cognitive index. The measure demonstrated good internal consistency (Cronbach’s α = 0.80; McDonald’s ω = 0.76), and a one-factor model indicated that all three assessments loaded strongly on a common latent factor (loadings ranging from 0.63 to 0.77). Taken together, these results suggest that the composite index captures a coherent and reliable latent construct of children’s cognitive functioning (The psychometric properties of the cognitive ability composite are reported in Supplementary Appendix 1). The resulting index was standardized to have a mean of zero and a standard deviation of one. As such, regression coefficients may be interpreted as changes in cognitive ability measured in standard-deviation units.

The cognitive measures used in this study have been employed in research on child development in Ghana. Digit Span tasks have been used to assess attention, working memory, and executive functioning, while Raven’s Progressive Matrices have demonstrated validity as a measure of non-verbal reasoning in Ghanaian populations (Anum, 2014, 2022; Augsburg et al., 2024).

### 2.2 Main explanatory variable

The key explanatory variable, household mental distress (HMD), measures the mental health status of adults residing in the child’s household. Following previous studies (Canavan et al., 2013; Owoo and Lambon-Quayefio, 2021), we assess mental distress using the Kessler Psychological Distress Scale (K10), a widely used screening instrument for non-specific psychological distress based on ten self-reported symptoms.^2^ In each household, the K10 questions were administered to the household head, one spouse (typically the first listed spouse when present), and an additional household member aged 12 years or older.

To construct HMD, we first aggregated individual K10 scores at the household level. For each household surveyed, the mean K10 score is calculated for all adult members aged 18 and above who provided valid responses. This yields a continuous mean K10 score for the household, which reflects the severity of mental distress experienced by its members. Following the classification guidelines proposed by Andrews and Slade (2001), K10 scores of 19 or below are generally interpreted as indicating a low likelihood of clinically meaningful psychological distress, whereas scores above 19 are associated with increasing levels of mild, moderate, or severe distress. Accordingly, households with an average adult K10 score of 19 or below are classified as not distressed and coded as 0, while households with an average score exceeding 19 are classified as distressed and coded as 1. We use this binary measure as our primary indicator of household mental distress because it facilitates interpretation and distinguishes households experiencing elevated levels of psychological distress from those that are not. To ensure that the results are not driven by this classification threshold, we re-estimate all models using the continuous household K10 score as a robustness check.

The K10 has been widely used and validated in research using data from Ghana (Canavan et al., 2013; Sipsma et al., 2013; Owoo and Lambon-Quayefio, 2021; Vitellozzi et al., 2024; Amenah et al., 2025).

### 2.3 Instrumental variable

In addition to household-level mental distress, we construct a measure of community-level mental distress (CMD) using information from all surveyed households within each Enumeration Area (EA).^3^ For each EA and survey wave, we calculate the proportion of households classified as experiencing mental distress. To avoid a mechanical correlation between a household’s distress status and the community average (Manski, 1993), we employ a leave-one-out (LOO) IV approach. Specifically, CMD is defined as the proportion of distressed households in an EA that excludes the household under consideration. For example, if ten households are surveyed within an EA, the CMD value for a given household is calculated using the distress status of the remaining nine households. Households located in EAs with only one sampled household are excluded from this calculation. Thus, the measure reflects the prevalence of psychological distress among other households in the same community. Since CDM varies across both space (EAs) and time (survey waves), it captures changes in the broader social and economic conditions households experience within the same locality. Similar leave-one-out community averages have been used as instrumental variables in previous studies (Mangyo, 2008; Lamichhane and Mangyo, 2011; Opoku et al., 2026).

### 2.4 Control variables

We include several child and household characteristics that may affect children’s cognitive ability as control variables. Specifically, we include both linear and quadratic terms of the child’s age and years of education to capture any non-linearities in the relationship between the children’s cognitive ability and these variables. We also include a binary indicator for the child’s gender. The household-level controls consist of the number of dependents, a binary indicator for area of residence (urban or rural), and a household wealth index to assess economic status. Controlling for the number of dependents is particularly important because it partly captures potential resource dilution within households and variations in caregiving demands that may influence children’s cognitive development. We note that our instrumental variable, community-level mental distress, may be influenced by neighborhood socioeconomic factors. We therefore include a leave-one-out measure of community wealth (CW). CW is calculated as the mean wealth of all other households within the same EA and survey wave, excluding the household in question. This measure reflects the average economic standing of households within the same community and survey period, helping to ensure that the identified association is less likely to be influenced by neighborhood-level deprivation or local resource limitations than by household mental distress. We include both linear and quadratic terms of the household and community-level wealth index to capture any nonlinear effects of wealth on children’s cognitive development.

### 2.5 Data and descriptive statistics

The dataset is an unbalanced panel, as many children and households do not appear in all waves. The combined sample includes 12,846 child-wave observations and 3,174 household-wave observations, with 8,755 unique children and 3,174 unique households. Among the children, roughly 62% appear in just one wave, 29% in two waves, and 9% in three waves. For households, approximately 32% are observed in a single wave, 35% in two waves, and 33% in three waves.

The ages of respondents to the cognitive assessment questions vary across waves. In Waves 1 and 2, the assessments were administered to children aged 5 to 15 years, whereas in Wave 3, the assessments were expanded to include all household members aged 5 years and older. To maximize sample size, we combined the data from all three waves and extended our analysis to children and adolescents aged 5 to 17. To ensure that our results are robust, we present findings for the entire sample (ages 5 to 17) and two subsamples: pre-teens (ages 5 to 12) and teens (ages 13 to 17).

Table 1 presents descriptive statistics for the full sample, as well as for the pre-teen and teen subsamples. As expected, cognitive performance differs across age groups. Teens exhibit higher average cognitive index scores than pre-teens, reflecting age-related cognitive maturation and accumulated educational experience. Mental distress is common among households within the sample, with approximately 40.5% of children residing in households classified as experiencing mild to severe psychological distress. This prevalence is similar across age groups, affecting 40.8% of pre-teens and 39.6% of adolescents. Community-level mental distress shows a consistent pattern, with an average of 41.5% of households in an EA classified as distressed. The similarity between household-level and community-level distress indicates a significant spatial clustering of mental health conditions. Supporting this interpretation, the correlation between household and community mental distress is approximately 0.46. The data also reveal a spatial clustering of economic wellbeing, with a correlation between household and community wealth of about 0.64. Both correlations are statistically significant at the 1% level.

**TABLE 1:** Summary statistics.

	Full sample		Pre-teen (5–12 years)		Teen (13–17 years)	
VARIABLES	Mean	SD	Mean	SD	Mean	SD
Cognitive ability	0.047	0.972	−0.158	0.899	0.525	0.969
Household mental distress	0.405	0.491	0.408	0.492	0.396	0.489
Household mental distress score	18.483	5.715	18.53	5.689	18.374	5.774
Community mental distress	0.415	0.222	0.42	0.227	0.405	0.211
Community mental distress score	18.692	2.882	18.757	2.938	18.542	2.742
Age (years)	10.316	3.305	8.585	2.243	14.343	1.199
Age squared	117.339	69.899	78.738	38.62	207.171	35.312
Gender–female	0.47	0.499	0.474	0.499	0.463	0.499
Education (years)	2.65	2.666	1.52	1.763	5.28	2.554
Education squared	14.13	21.015	5.42	9.129	34.4	26.233
Number of dependents	3.471	1.838	3.617	1.802	3.131	1.877
Urban residence	0.286	0.452	0.276	0.447	0.308	0.462
Household wealth	1.791	0.986	1.771	0.985	1.838	0.988
Household wealth squared	4.18	6.553	4.105	6.658	4.354	6.298
Community wealth	1.865	0.591	1.856	0.601	1.884	0.566
Community wealth squared	3.826	4.357	3.806	4.521	3.872	3.949
Observations	12,846		8,985		3,861	

The sample’s gender distribution is fairly balanced, with females accounting for 47% of all observations. The typical child is 10.3 years old and has about 2.6 years of schooling. On average, households have 3.5 dependents, and about 29% of children live in urban areas.

## 3 Estimation

Following the human capital production framework (Becker and Tomes, 1986; Cunha and Heckman, 2007), we model child cognitive ability as a function of household conditions, individual characteristics, and environmental factors. To examine the relationship between household mental distress and child cognitive ability, we estimate (Equation 1) using a pooled ordinary least squares (OLS) specification with survey-wave and region fixed effects:


$$C\,o\,g\,A_{i\,h\,c\,r\,t}=\beta_{0}+\beta_{1}\,H\,M\,D_{h\,c\,r\,t}+\beta_{2}\,C\,W_{-h,c\,t}+\beta_{3}\,X_{i\,h\,c\,r\,t}$$ (1)

$$\;\;+\lambda_{t}+\delta_{r}+\varepsilon_{i\,h\,c\,r\,t}$$

where *i*, *h*, *c*, *t*, and *r* denote child, household, community, survey wave, and region, respectively. The dependent variable, *CogA*_*ihct*_, represents the cognitive ability index of child *i*; *HMD*_*hct*_ is a binary indicator equal to one if the household is mentally distressed; and *CW*_−*h*,*ct*_ denotes the leave-one-out mean community wealth in community *c* and survey wave t, excluding household h. *X*_*ihct*_ represents a vector of child and household-level controls, while λ_*t*_, and δ_*r*_ denote survey-wave and region fixed effects, respectively. Standard errors are clustered at the household level to allow for correlation among children from the same household. The parameter of interest, β_1_, captures the average difference in cognitive ability between children living in mentally distressed and non-distressed households, conditional on household, community, and regional characteristics.

### 3.1 Instrumental variable

Household mental distress may be endogenous because of reverse causality, omitted variables, and measurement error. Psychological distress within the household may reduce parental investments, emotional support, and the quality of the home learning environment, all of which are important determinants of children’s cognitive development (Cunha and Heckman, 2007; Farah et al., 2008; Lehrl et al., 2020). Conversely, children experiencing developmental difficulties may increase caregiving demands and psychological stress among household members, generating the potential for simultaneity bias (Mensah and Kiernan, 2010; Wolicki et al., 2021). To address these concerns, we employ a two-stage least squares (2SLS) instrumental variable (IV) approach, using a leave-one-out measure of community mental distress to instrument household mental distress. The first- and second-stage specifications are given by Equations 2,3, respectively:

First stage:


$$H\,M\,D_{h\,c\,r\,t}=\pi_{0}+\pi_{1}\,C\,M\,D_{-h,c\,t}+\pi_{2}\,C\,W_{-h,c\,t}+\pi_{3}\,X_{i\,h\,c\,r\,t}$$ (2)

$$\;\;+\lambda_{t}+\delta_{r}+u_{h\,c\,r\,t}$$

Second stage:


$$C\,o\,g\,A_{i\,h\,c\,r\,t}=\alpha_{0}+\alpha_{1}\,{\hat{H\,M\,D}}_{h\,c\,r\,t}+\alpha_{2}\,C\,W_{-h,c\,t}+\alpha_{3}\,X_{i\,h\,c\,r\,t}$$ (3)

$$\;\;+\lambda_{t}+\delta_{r}+\eta_{i\,h\,c\,r\,t}$$

Where *CMD*_−*h*,*ct*_ is the average mental health of households in community c, excluding household h, and *CW*_−*h*,*ct*_ is the corresponding leave-one-out measure of community wealth.

We argue that households residing in the same community are exposed to a shared psychological and social environment. Social interactions, emotional contagion, and common exposure to local stressors may contribute to the clustering of mental distress within communities (Kawachi and Berkman, 2001; Ross, 2000; Kim et al., 2008). Consequently, community-level mental distress is expected to be strongly associated with household mental distress.

A key concern is that community-level mental distress may proxy for broader community characteristics that directly influence child cognitive development, such as neighborhood deprivation, infrastructure quality, school quality, or access to local resources. To address this concern, all specifications include a LOO measure of community wealth, survey-wave fixed effects, and region fixed effects. The inclusion of a LOO measure of community wealth is particularly important because it helps distinguish the effect of community mental distress from broader socioeconomic conditions. By controlling for the average wealth of other households in the community, the estimates are less likely to reflect neighborhood deprivation, local resource constraints, or community-level economic disadvantage that may independently influence children’s cognitive development. Importantly, the inclusion of the LOO measure of community wealth allows us to distinguish the influence of the broader socioeconomic environment from that of community mental distress. The estimated effect of household mental distress remains substantively unchanged after accounting for community wealth, suggesting that neighborhood economic conditions alone are unlikely to explain the observed relationship. We also note that cognitive assessments like digit span and Raven’s matrices primarily measure working memory and fluid reasoning, which are more closely linked to the home learning environment than to short-term community-level shocks (Farah et al., 2008).^4^ In addition, survey-wave fixed effects absorb common national shocks and temporal trends, while region fixed effects account for persistent regional differences in economic development, educational infrastructure, institutional quality, and public service provision. Identification, therefore, arises from within-region variation in community mental distress over time rather than from broad geographic disparities. Furthermore, Lewbel (2012) estimates and the placebo tests described below provide additional support for the validity of our identification strategy and reduce concerns that the estimated relationship is driven by spurious correlations.

### 3.2 Robustness check

We supplement the IV approach with four additional sensitivity analyses. First, we assess the robustness of our 2SLS estimates using the heteroskedasticity-based identification strategy proposed by Lewbel (2012). Unlike our primary analysis, which relies on community-level mental distress as an external source of variation, the Lewbel method exploits patterns in the data itself to generate additional instruments. Because the Lewbel approach relies on a different source of identification than our primary instrument, obtaining comparable estimates would provide additional evidence that the main findings are not driven by the specific choice of instrument.

Second, our primary analysis defines household mental distress using a binary indicator based on established K10 classification thresholds. Although this approach facilitates interpretation and is consistent with previous studies, it may obscure meaningful variation in the severity of psychological distress across households. To assess whether our findings depend on the chosen classification threshold, we re-estimate the IV model using the continuous household mental distress score rather than the binary indicator. Additionally, this approach allows us to examine whether there is a linear-dose relationship between household mental distress and child cognitive ability.

Third, we employ two complementary treatment-effects estimators: propensity score matching (PSM) and inverse probability weighted regression adjustment (IPWRA). These approaches address potential selection bias arising from observable differences between children residing in distressed and non-distressed households. Specifically, the PSM constructs a matched comparison group of children with similar observed characteristics using nearest-neighbor matching, while IPWRA combines propensity score weighting with outcome regression adjustment, providing doubly robust estimates when either the treatment or outcome model is correctly specified.

Finally, we perform a randomization-inference (RI) placebo test, following the approach of Jose and Younas (2023), by randomly assigning treatment status and comparing the observed treatment effect against the distribution of placebo effects. If the observed effect falls in the extreme tail, it suggests that our findings are unlikely due to random chance. Consistent results across these methods enhance our confidence in our findings.

## 4 Results

This section presents and discusses the results in three subsections. First, the results from the benchmark pooled OLS regressions and the main 2SLS estimates are presented, followed by robustness checks.

### 4.1 Benchmark pooled OLS estimates

Table 2 reports the results from the pooled OLS regressions. Columns 1–3 present estimates without community-level wealth, and columns 4–6 include community wealth. Across all specifications, household mental distress is negatively and significantly associated with children’s cognitive outcomes.

**TABLE 2:** Benchmark pooled OLS estimates.

	Full	Pre-teen	Teen	Full	Pre-teen	Teen
VARIABLES	(1)	(2)	(3)	(4)	(5)	(6)
Household mental distress	−0.079***	−0.077***	−0.080***	−0.081***	−0.079***	−0.082***
	(0.019)	(0.021)	(0.031)	(0.019)	(0.021)	(0.031)
Age (years)	0.255***	0.297***	0.712***	0.255***	0.295***	0.717***
	(0.015)	(0.027)	(0.275)	(0.015)	(0.027)	(0.275)
Age squared	−0.011***	−0.014***	−0.027***	−0.011***	−0.014***	−0.027***
	(0.001)	(0.002)	(0.009)	(0.001)	(0.002)	(0.009)
Gender–female	−0.059***	−0.048***	−0.081***	−0.060***	−0.048***	−0.082***
	(0.014)	(0.015)	(0.027)	(0.014)	(0.015)	(0.028)
Education (years)	0.230***	0.217***	0.214***	0.229***	0.216***	0.212***
	(0.011)	(0.017)	(0.020)	(0.011)	(0.017)	(0.020)
Education squared	−0.009***	−0.005*	−0.007***	−0.009***	−0.005*	−0.006***
	(0.001)	(0.003)	(0.002)	(0.001)	(0.003)	(0.002)
Number of dependents	−0.018***	−0.022***	−0.008	−0.018***	−0.022***	−0.008
	(0.005)	(0.006)	(0.009)	(0.005)	(0.006)	(0.009)
Urban residence	0.142***	0.123***	0.168***	0.130***	0.108***	0.165***
	(0.025)	(0.027)	(0.044)	(0.026)	(0.027)	(0.045)
Household wealth	0.161***	0.183***	0.103***	0.174***	0.185***	0.146*
	(0.024)	(0.026)	(0.040)	(0.041)	(0.043)	(0.078)
Household wealth squared	−0.015***	−0.017***	−0.012**	−0.020**	−0.018*	−0.022
	(0.003)	(0.004)	(0.006)	(0.009)	(0.009)	(0.018)
Community wealth				0.108	0.151*	−0.029
				(0.076)	(0.081)	(0.136)
Community wealth squared				−0.006	−0.012	0.013
				(0.013)	(0.013)	(0.027)
Constant	−1.856***	−2.039***	−5.055**	−2.058***	−2.280***	−5.127**
	(0.083)	(0.119)	(1.994)	(0.130)	(0.161)	(1.998)
Observations	12,846	8,985	3,861	12,846	8,985	3,861
R-squared	0.406	0.381	0.262	0.407	0.382	0.262
Survey year FE	Yes	Yes	Yes	Yes	Yes	Yes
Region FE	Yes	Yes	Yes	Yes	Yes	Yes

Cluster-robust standard errors in parentheses, clustered at the household level.

***p < 0.01,

**p < 0.05,

*p < 0.1.

In the full sample (column 1), children living in mentally distressed households score approximately 0.079 standard deviations below those living in non-distressed households, holding constant child, household, and regional characteristics. Similar effects are observed among pre-teens and teenagers, with estimated coefficients ranging from −0.077 to −0.082 standard deviations. These findings suggest that household mental distress is consistently associated with lower cognitive ability across age groups.

The results in columns (4) to (6) indicate that there is no significant relationship between community wealth and children’s cognitive abilities after controlling for child, household, and regional characteristics. Furthermore, comparing columns (1) to (3) with columns (4) to (6) shows that including the CW measure has little effect on the estimated HMD coefficient. The size and statistical significance of this coefficient remain largely unchanged across all samples. While community wealth cannot capture all neighborhood characteristics, the stability of the estimates after its inclusion suggests that the results are not primarily driven by observable community-level socioeconomic conditions. These findings provide preliminary support for the notion that household mental distress is independently linked to children’s cognitive development.

The control variables mostly show expected relationships with cognitive ability. Age correlates positively with cognitive performance, but the rate of improvement slows with increasing age. Educational attainment is positively correlated with cognitive ability, though the benefit lessens with each extra year of schooling. On average, girls score lower than boys, while children in urban areas tend to have notably higher cognitive scores than those in rural areas. Household wealth is also positively associated with cognitive ability, but the gains decrease at higher wealth levels. The number of dependents in the household is negatively associated with children’s cognitive ability in the full sample and among pre-teens, providing evidence consistent with resource dilution within larger households. Specifically, each additional dependent is associated with a reduction of approximately 0.018 to 0.022 standard deviations in children’s cognitive ability. In contrast, the coefficient is small and not statistically significant among teenagers, suggesting that the dependency burden may be more consequential for younger children.

### 4.2 Instrumental variable estimates

Table 3 presents 2SLS IV estimates of the relationship between household mental distress and children’s cognitive ability. The first-stage results indicate that the instrument is relevant. Community-level mental distress is positive and statistically significant at the 1% level across all specifications, with coefficients ranging from 0.893 to 0.906. Moreover, the Kleibergen–Paap rk Wald F-statistics range from 537.2 to 904.5, far exceeding the conventional threshold of 10 and the more stringent thresholds proposed by Stock and Yogo (2002). These statistics rule out weak instrument concerns and support the instrument’s strong predictive power.

**TABLE 3:** IV estimates—main model (external instrument).

	Full	Pre-teen	Teen
VARIABLES	(1)	(2)	(3)
Household mental distress	−0.468***	−0.476***	−0.429***
	(0.054)	(0.056)	(0.092)
First stage			
Community mental distress	0.898***	0.893***	0.906***
	(0.030)	(0.032)	(0.039)
KP rk Wald F	904.5	781.8	537.2
Observations	12,846	8,985	3,861
R-squared	0.372	0.339	0.234
Control variables	Yes	Yes	Yes
Survey year FE	Yes	Yes	Yes
Region FE	Yes	Yes	Yes

Cluster-robust standard errors in parentheses, clustered at the household level.

***p < 0.01. The 2SLS specification uses community-level mental distress as the external instrument.

The second-stage estimates show that across all specifications, household mental distress is associated with substantial and statistically significant reductions in children’s cognitive ability. For the full sample, children living in households experiencing mental distress score approximately 0.47 standard deviations lower on the cognitive ability index than comparable children in non-distressed households ($\hat{\alpha_{1}}$ = −0.468, *p* < 0.01). The estimated effect is similarly large across the two age groups, though slightly higher for pre-teens ($\hat{\alpha_{1}}$ = −0.476, *p* < 0.01) and for adolescents aged 13–17 years ($\hat{\alpha_{1}}$ = −0.429, *p* < 0.01).

Note that the IV estimates are considerably larger than the corresponding OLS estimates reported in Table 2. Whereas the OLS estimates suggest cognitive deficits of about 0.08 standard deviations, the IV estimates imply effects roughly five to six times larger. This pattern is consistent with attenuation bias from measurement error in household mental distress, or with other sources of endogeneity that bias the OLS estimates toward zero. The results, therefore, suggest that conventional regression methods may substantially underestimate the adverse consequences of household mental distress for children’s cognitive outcomes.

### 4.3 Robustness and sensitivity checks

In this section, we conduct a series of robustness and sensitivity checks to assess the consistency of our findings. We begin with the Lewbel IV estimates, then the IV model with the continuous HMD score as the outcome, followed by the PSM and IPWRA estimates, and finally the results from our RI placebo test.

Table 4 presents the results from the Lewbel IV estimates. The estimates remain qualitatively similar to those from the 2SLS estimates in Table 3.

**TABLE 4:** IV estimates—robustness (external IV + Lewbel).

	Full	Pre-teen	Teen
VARIABLES	(1)	(2)	(3)
Household mental distress	−0.327***	−0.349***	−0.223***
	(0.050)	(0.054)	(0.080)
First stage			
Community mental distress	1.015***	1.009***	1.032***
	(0.048)	(0.051)	(0.066)
KP rk Wald F	45.02	38.43	25.80
Hansen J	102.3	80.35	64.35
Observations	12,846	8,985	3,861
R-squared	0.393	0.362	0.258
Controls variables	Yes	Yes	Yes
Survey year FE	Yes	Yes	Yes
Region FE	Yes	Yes	Yes

Cluster-robust standard errors in parentheses, clustered at the household level. The Lewbel+ External IV specification combines the external instrument (CMD) with the Lewbel-generated instruments based on heteroskedasticity.

***p < 0.01.

Household mental distress is associated with a 0.33 standard deviation reduction in cognitive ability ($\hat{\alpha_{1}}$ = −0.327, *p* < 0.01) for the full sample. Comparable effects are observed among pre-teens ($\hat{\alpha_{1}}$ = −0.349, *p* < 0.01) and teens ($\hat{\alpha_{1}}$ = −0.223, *p* < 0.01). Although the Lewbel estimates are smaller in magnitude than the baseline 2SLS estimates, they remain economically meaningful and statistically significant. The first-stage estimates remain strong, and the Kleibergen–Paap rk Wald F statistics range from 25.8 to 45.0, comfortably exceeding conventional weak-instrument thresholds.

Table 5 presents the results of a 2SLS analysis in which the binary HMD indicator is replaced with a continuous HMD score. The results are qualitatively similar to the 2SLS estimates in Table 3. The first-stage estimates show a strong relationship between community-level and household-level mental distress across all samples. The second-stage results are consistent with the main findings. Specifically, a one-unit increase in the HMD score is associated with declines of approximately 0.030 to 0.035 standard deviations in cognitive ability across these samples. The standard deviation of the HMD score in the full sample is approximately 5.7 points (see Table 1). This means that a one-standard-deviation increase in the HMD score is associated with a reduction of about 0.20 standard deviations in children’s cognitive ability.

**TABLE 5:** IV estimates—continuous measure of HMD (external instrument).

	Full	Pre-teen	Teen
VARIABLES	(1)	(2)	(3)
Household mental distress score	−0.034***	−0.035***	−0.030***
	(0.004)	(0.005)	(0.008)
First stage			
Community mental distress score	0.854***	0.851***	0.859***
	(0.029)	(0.031)	(0.038)
KP rk Wald F	857.3	733.9	497.6
Observations	12,846	8,985	3,861
R-squared	0.387	0.356	0.250
Control variables	Yes	Yes	Yes
Survey year FE	Yes	Yes	Yes
Region FE	Yes	Yes	Yes

Cluster-robust standard errors in parentheses, clustered at the household level,

***p < 0.01.

Table 6 presents the PSM and IPWRA estimates. Our main results hold across both estimators and samples. The PSM estimates indicate that children in distressed households score about 0.076 to 0.089 standard deviations lower on the cognitive ability index than similar children in non-distressed households. The IPWRA estimates show that household mental distress is associated with a 0.067 to 0.073 standard deviation reduction in cognitive ability. We note that the PSM and IPWRA estimates are comparable in magnitude to the benchmark pooled OLS results in Table 2 but smaller than the main 2SLS estimates in Table 3. One possible explanation is that the treatment-effects estimators adjust for differences in observed characteristics between households, whereas the IV approach is designed to address potential endogeneity arising from unobserved confounding and measurement error.

**TABLE 6:** PSM and IPWRA estimates.

	PSM			IPWRA		
	Full	Pre-teen	Teen	Full	Pre-teen	Teen
VARIABLES	(1)	(2)	(3)	(4)	(5)	(6)
Household mental distress	−0.076***	−0.078***	−0.089**	−0.069***	−0.073***	−0.067**
	(0.019)	(0.021)	(0.040)	(0.020)	(0.022)	(0.031)
Observations	12,846	8,985	3,861	12,846	8,985	3,861
Controls variables	Yes	Yes	Yes	Yes	Yes	Yes
Survey year FE	Yes	Yes	Yes	Yes	Yes	Yes
Region FE	Yes	Yes	Yes	Yes	Yes	Yes
Matching estimator	1-to-1	1-to-1	1-to-1	IPWRA	IPWRA	IPWRA
Estimand	ATE	ATE	ATE	ATE	ATE	ATE

Robust standard errors in parentheses.

***p < 0.01,

**p < 0.05.

Finally, Table 7 presents the results of the RI placebo test. Out of 600 placebo iterations, only a small number of simulated coefficients reach conventional levels of statistical significance. For the full sample, the number of significant placebo estimates is limited (68, 38, and 21 across different thresholds), while the maximum placebo t-statistic (2.929) remains below the absolute value of the original estimate (−4.185). Similar patterns are observed for the pre-teen and teen samples. The non-parametric *p*-values further confirm the robustness of the findings, with values of 0.000 for the full sample and pre-teen group, and 0.008 for the teen group. These results indicate that the likelihood of obtaining the observed estimates by random chance is extremely low.

**TABLE 7:** Randomization-inference placebo test results.

	|t| > 1.645	t > 1.645	t > 1.960	Max t (placebo)	t (original)
SAMPLES	(1)	(2)	(3)	(4)	(5)
Full sample	68	38	21	2.929	−4.185
Pre-teen	66	36	20	2.61	−3.738
Teen	56	34	19	3.305	−2.649

Columns (1)–(3) report the number of iterations (out of 600) in which the placebo coefficient attained the significance level indicated by the column heading. Column (4) is the maximum t-statistic recorded across all 600 placebo regressions. Column (5) is the t-statistic from the main regression in Table 3. The placebo treatment is constructed by randomly permuting household-level mental distress across households within each survey wave. Non-parametric *p*-values (share of iterations where |placebo t|≥|original t|): full sample *p* = 0.000; pre-teen *p* = 0.000; teen *p* = 0.008.

Overall, the Lewbel IV specification, the IV using the continuous measure of HMD, the PSM and IPWRA estimates, and placebo tests all yield the same conclusion: children living in households experiencing mental distress show significantly lower cognitive ability. The consistency of these findings across multiple estimation strategies strengthens confidence in the robustness of the main results.

## 5 Discussion

This study examined the relationship between household mental distress and children’s cognitive abilities using nationally representative longitudinal data from Ghana. We found a significant association between household mental distress across the full sample, the pre-teen sample, and the teen sample. The IV estimates indicate that children living in mentally distressed households score approximately 0.43 to 0.48 standard deviations lower on the cognitive ability index than comparable children living in non-distressed households.

The results indicate that the effects of household psychological conditions extend beyond early childhood and remain relevant during later developmental stages marked by significant cognitive, social, and emotional changes. Additionally, the findings are consistent with previous research that highlights negative impacts of caregiver mental health on children’s cognitive and socio-emotional development (Bennett et al., 2016; Leijdesdorff et al., 2017; Wolicki et al., 2021). Notably, this study emphasizes the broader household psychological environment, rather than individual caregivers alone, as an important factor affecting children, particularly in developing countries where caregiving roles are often shared among multiple adults.

To contextualize the magnitude of the estimated effects, it is useful to compare them with those reported in related studies. For example, parental job loss in the United States has been associated with reductions in children’s test scores of approximately 0.3 to 0.5 standard deviations (Lindo, 2011), while parental depression has been linked to cognitive deficits ranging from 0.25 to 0.45 standard deviations in high-income countries (Mensah and Kiernan, 2010). The estimated effects in this study are broadly comparable in magnitude, suggesting that household mental distress may represent an important developmental risk factor for children in Ghana.

Additionally, the results using a continuous measure of household mental distress indicate that the association with children’s cognitive outcomes extends beyond a simple distressed/non-distressed distinction. Specifically, higher levels of household psychological distress are associated with poorer cognitive outcomes, suggesting that the relationship is not limited to households experiencing severe distress. This pattern is consistent with evidence that psychological distress varies along a continuum rather than representing a binary condition (Kessler et al., 2002).

Several developmental and psychological perspectives provide potential explanations for the observed relationship. In many Ghanaian households, caregiving responsibilities are shared among multiple adults. Consequently, disruptions in household functioning may affect children’s developmental experiences through a broader set of relationships than those typically considered in studies focused primarily on the mental health of individual caregivers. Family stress models suggest that adult psychological distress can disrupt family functioning, decrease emotional availability, and diminish the quality of interactions between caregivers and children (Conger et al., 2002; Masarik and Conger, 2017). Distressed adults may have fewer cognitive and emotional resources to support children’s learning, supervise educational activities, or provide cognitively stimulating home environments. Likewise, family-systems perspectives posit that psychological difficulties experienced by one household member can influence the entire household’s functioning, thereby indirectly affecting children’s developmental experiences through changes in family relationships and household dynamics (Bowen, 1993; Cox and Paley, 1997; Goodman and Gotlib, 1999). These perspectives suggest that the effects of psychological distress may extend beyond the individual experiencing distress and influence the broader family environment in which children develop. Although this study does not directly test these mechanisms, the findings are consistent with the broader proposition that children’s cognitive development is embedded in and influenced by the household psychosocial environment.

### 5.1 Limitations and future research

Several limitations must be considered when interpreting these findings. First, the study relies on an instrumental variable strategy, which depends on the assumptions of instrument relevance and exogeneity. Although the leave-one-out community measure of mental distress predicts household mental distress strongly, the exclusion restriction cannot be directly tested. While we control for various household and community characteristics and conduct several robustness tests, unobserved community-level factors may still influence children’s cognitive development. Thus, any causal interpretation should be made with caution because it relies on the assumption that community mental distress affects children’s cognitive outcomes only through its effect on household mental distress.

Second, the GSPS does not identify primary caregiver–child relationships, caregiving responsibilities, or detailed household structures. Consequently, our measure of household mental distress captures the broader psychological environment by averaging K10 scores across all adult household members rather than focusing specifically on the mental health of primary caregivers. While this approach is appropriate for examining the overall household context, it may mask important differences within households. For example, the mental health of primary caregivers may have a stronger influence on children’s cognitive development than that of other adults, and the effects of household distress may vary depending on family structure, caregiving arrangements, or the roles that siblings play within the household. Future research with more detailed data on caregiving roles, family structure, and intra-household relationships could provide a deeper understanding of the relationship between household mental distress and children’s cognitive development.

Third, we do not formally test whether the cognitive index is measured equivalently across survey waves. Although the same cognitive assessments were administered using standardized procedures in each survey round, it remains possible that the underlying construct is not captured in the same way over time. Future research could examine the longitudinal measurement properties of the cognitive index using formal invariance tests.

Lastly, the GSPS panel is highly unbalanced, with many children observed only in a single survey wave. While this does not affect the use of the available observations, it limits our ability to follow the same children over time and examine individual developmental trajectories. As a result, the analysis primarily captures differences across children rather than changes within children as they age. Future studies using more balanced longitudinal data could help clarify how household mental distress influences cognitive development throughout childhood and adolescence.

## 6 Conclusion

This study provides evidence that children living in mentally distressed households exhibit significantly lower cognitive ability than their peers, with effects that persist across both pre-teens and teenagers. Unlike prior research that focuses on individual caregivers, this study conceptualizes mental distress as a household-level phenomenon, reflecting the shared caregiving arrangements common in Ghana and similar developing-country settings. This framing advances understanding of how the broader family psychosocial environment shapes children’s cognitive development. The findings suggest that efforts to improve children’s developmental outcomes may benefit from greater attention to adult mental health within the household, reinforcing the interconnected nature of family wellbeing and child development.

## Data Availability

Publicly available datasets were analyzed in this study. This data can be found here: https://egc.yale.edu/isser-northwestern-yale-long-term-ghana-socioeconomic-panel-survey-gsps.

## Footnotes

1 Raven’s Pattern Recognition Assessment is a 12-item adaptation of Raven’s Colored and Standard Progressive Matrices (CPM/SPM), which are widely recognized non-verbal measures of fluid intelligence (Raven, 2000). Research indicates that shorter versions of the Raven’s tests can accurately predict scores from the full version. For example, Langener et al. (2022) developed a 15-item short form that demonstrated correlations of *r* = 0.89–0.93 with the full 60-item version. These shorter forms have been extensively used in large-scale developmental surveys conducted in low- and middle-income countries.

2 The K10 is a widely used screening instrument that assesses psychological distress experienced during the previous four weeks. It consists of ten questions measuring symptoms of anxiety and depression, such as nervousness, hopelessness, restlessness, and feelings of worthlessness. Scores range from 10 to 50, with higher values indicating greater psychological distress.

3 The GSPS sampling structure groups households within geographically defined EAs, which represent small local communities, enabling us to aggregate mental health conditions at the community and survey-wave levels.

4 Farah et al. (2008) demonstrate that it is the quality of the home environment, rather than broader community conditions, that shapes memory-related cognitive capacities.

## References

[B1] Amenah M. A. Fenny A. Akazili J. Mirzoev T. Agyepong I. A. Mason T. (2025). Estimating the prevalence, socioeconomic determinants, and health seeking behavior of individuals with depression in Ghana. *Sci. Rep.* 15:22239. 10.1038/s41598-025-06134-2 40596148 PMC12215751

[B2] Andrews G. Slade T. (2001). Interpreting scores on the Kessler Psychological Distress Scale (K10). *Aust. N. Z. J. Public Health* 25 494–497. 10.1111/j.1467-842x.2001.tb00310.x 11824981

[B3] Annim S. K. Awusabo-Asare K. Amo-Adjei J. (2015). Household nucleation, dependency and child health outcomes in ghana. *J. Biosoc. Sci.* 47 565–592. 10.1017/S0021932014000340 25167165

[B4] Anum A. (2022). Does socio-economic status have different impact on fluid and crystallized abilities? comparing scores on Raven’s progressive matrices, kaufman assessment battery for children II story completion and kilifi naming test among children in ghana. *Front. Psychol.* 13:880005. 10.3389/fpsyg.2022.880005 35707655 PMC9189400

[B5] Anum A. (2014). A standardisation study of the Raven’s coloured progressive matrices in ghana. *IFE Psychol. Intern. J.* 22 27–35.

[B6] Augsburg B. Anamuah-Mensah J. Bornstein M. H. Krutikova S. Veroux O. A. Wolf S. (2024). *Cognition in Young Children in a Low-Income Rural Setting: Prominent Correlates in Sub-Saharan Ghanaian Preschoolers.* Oxford: Thrive, Oxford Policy Management.

[B7] Becker G. S. Tomes N. (1986). Human capital and the rise and fall of families. *J. Lab. Econ.* 4 1–47. 10.1086/298118 12146356

[B8] Bennett I. M. Schott W. Krutikova S. Behrman J. R. (2016). Maternal mental health, and child growth and development, in four low-income and middle-income countries. *J. Epidemiol. Commun. Health* 70 168–173. 10.1136/jech-2014-205311 26359503 PMC5392254

[B9] Blakemore S. J. (2019). Adolescence and mental health. *Lancet* 393 2030–2031. 10.1016/S0140-6736(19)31013-X 31106741

[B10] Bowen M. (1993). *Family Therapy in Clinical Practice.* London: Bloomsbury Publishing.

[B11] Canavan M. E. Sipsma H. L. Adhvaryu A. Ofori-Atta A. Jack H. Udry C. et al. (2013). Psychological distress in Ghana: Associations with employment and lost productivity. *Int. J. Ment. Health Syst.* 7:9. 10.1186/1752-4458-7-9 23497536 PMC3599820

[B12] Conger R. D. Wallace L. E. Sun Y. Simons R. L. McLoyd V. C. Brody G. H. (2002). Economic pressure in African American families: A replication and extension of the family stress model. *Dev. Psychol.* 38 179–193. 10.1037/0012-1649.38.2.179 11881755

[B13] Cox M. J. Paley B. (1997). Families as systems. *Annu. Rev. Psychol.* 48 243–267. 10.1146/annurev.psych.48.1.243 9046561

[B14] Cunha F. Heckman J. J. (2007). The technology of skill formation. *Am. Econ. Rev.* 97 31–47. 10.1257/aer.97.2.31

[B15] Diamond A. (2013). Executive functions. *Annu. Rev. Psychol.* 64 135–168. 10.1146/annurev-psych-113011-143750 23020641 PMC4084861

[B16] Duncan G. J. Dowsett C. J. Claessens A. Magnuson K. Huston A. C. Klebanov P. et al. (2007). School readiness and later achievement. *Dev. Psychol.* 43 1428–1446. 10.1037/0012-1649.43.6.1428 18020822

[B17] Farah M. J. Betancourt L. Shera D. M. Savage J. H. Giannetta J. M. Brodsky N. L. et al. (2008). Environmental stimulation, parental nurturance and cognitive development in humans. *Dev. Sci.* 11 793–801. 10.1111/j.1467-7687.2008.00688.x 18810850

[B18] Goodman S. H. Gotlib I. H. (1999). Risk for psychopathology in the children of depressed mothers: A developmental model for understanding mechanisms of transmission. *Psychol. Rev.* 106 458–490. 10.1037/0033-295x.106.3.458 10467895

[B19] Heckman J. J. (2006). Skill formation and the economics of investing in disadvantaged children. *Science* 312 1900–1902. 10.1126/science.1128898 16809525

[B20] Howard A. H. Dadirai Gwenzi G. Newsom L. Gebru B. T. Gilbertson Wilke N. (2023). The relationship between sense of belonging and well-being outcomes in emerging adults with care experience. *Int. J. Environ. Res. Public Health* 20:6311. 10.3390/ijerph20136311 37444158 PMC10341974

[B21] Huhtala M. Korja R. Lehtonen L. Haataja L. Lapinleimu H. Rautava P. (2014). Associations between parental psychological well-being and socio-emotional development in 5-year-old preterm children. *Early Hum. Dev.* 90 119–124. 10.1016/j.earlhumdev.2013.12.009 24418104

[B22] Jose J. Younas J. (2023). Financial inclusion and women’s bargaining power: Evidence from India. *Intern. Rev. Appl. Econ.* 37 76–92. 10.1080/02692171.2022.2044459

[B23] Kawachi I. Berkman L. F. (2001). Social ties and mental health. *J. Urban Health* 78 458–467. 10.1093/jurban/78.3.458 11564849 PMC3455910

[B24] Kessler R. C. Andrews G. Colpe L. J. Hiripi E. Mroczek D. K. Normand S. L. et al. (2002). Short screening scales to monitor population prevalences and trends in non-specific psychological distress. *Psychol. Med.* 32 959–976. 10.1017/s0033291702006074 12214795

[B25] Kim H. S. Sherman D. K. Taylor S. E. (2008). Culture and social support. *Am. Psychol.* 63 518–526. 10.1037/0003-066X 18793039

[B26] Kramer M. Kumar Gautam S. Vollmer S. (2021). Improving child health and cognition: Evidence from a school-based nutrition intervention in India. *Rev. Econ. Statist.* 103 818–834. 10.1162/rest_a_00906

[B27] Lamichhane D. K. Mangyo E. (2011). Water accessibility and child health: Use of the leave-out strategy of instruments. *J. Health Econ.* 30 1000–1010. 10.1016/j.jhealeco.2011.07.001 21802755

[B28] Langener A. M. Kramer A. W. van den Bos W. Huizenga H. M. A. (2022). shortened version of Raven’s standard progressive matrices for children and adolescents. *Br. J. Dev. Psychol.* 40 35–45. 10.1111/bjdp.12381 34041776 PMC9290746

[B29] Lehrl S. Evangelou M. Sammons P. (2020). The home learning environment and its role in shaping children’s educational development. *School Effect. School Improvement* 31 1–6. 10.1080/09243453.2020.1693487

[B30] Leijdesdorff S. van Doesum K. Popma A. Klaassen R. van Amelsvoort T. (2017). Prevalence of psychopathology in children of parents with mental illness and/or addiction: An up to date narrative review. *Curr. Opin. Psychiatry* 30 312–317. 10.1097/YCO.0000000000000341 28441171

[B31] Lewbel A. (2012). Using heteroscedasticity to identify and estimate mismeasured and endogenous regressor models. *J. Bus. Econ. Statist.* 30 67–80. 10.1080/07350015.2012.643126

[B32] Lindo J. M. (2011). Parental job loss and infant health. *J. Health Econ.* 30 869–879. 10.1016/j.jhealeco.2011.06.008 21798606

[B33] Luby J. Belden A. Botteron K. Marrus N. Harms M. P. Babb C. et al. (2013). The effects of poverty on childhood brain development: The mediating effect of caregiving and stressful life events. *JAMA Pediatr.* 167 1135–1142. 10.1001/jamapediatrics.2013.3139 24165922 PMC4001721

[B34] Mangyo E. (2008). The effect of water accessibility on child health in China. *J. Health Econ.* 27 1343–1356. 10.1016/j.jhealeco.2008.04.004 18511138

[B35] Manski C. F. (1993). Identification of endogenous social effects: The reflection problem. *Rev. Econ. Stud.* 60 531–542. 10.2307/2298123

[B36] Masarik A. S. Conger R. D. (2017). Stress and child development: A review of the Family Stress Model. *Curr. Opin. Psychol.* 13 85–90. 10.1016/j.copsyc.2016.05.008 28813301

[B37] Mensah F. K. Kiernan K. E. (2010). Parents’ mental health and children’s cognitive and social development: Families in England in the Millennium Cohort Study. *Soc. Psychiatry Psychiatr. Epidemiol.* 45 1023–1035. 10.1007/s00127-009-0137-y 19823757

[B38] Opoku A. Avorkpo E. A. Twumasi A. D. Sebu J. Nunoo J. Kofinti R. E. (2026). Non-farm entrepreneurship and household economic precarity in rural Ghana. *Rev. Dev. Econ.* 30 395–410. 10.1111/rode.13138

[B39] Owoo N. S. Lambon-Quayefio M. P. (2021). Mixed methods exploration of Ghanaian women’s domestic work, childcare and effects on their mental health. *PLoS One* 16:e0245059. 10.1371/journal.pone.0245059 33529183 PMC7853525

[B40] Raven J. (2000). The Raven’s Progressive Matrices: Change and Stability Over Culture and Time. *Cogn. Psychol.* 41, 1–48. 10.1006/cogp.1999.0735 10945921

[B41] Ross C. E. (2000). Neighborhood disadvantage and adult depression. *J. Health Soc. Behav.* 41 177–187. 10.2307/2676304

[B42] Sipsma H. Ofori-Atta A. Canavan M. Osei-Akoto I. Udry C. Bradley E. H. (2013). Poor mental health in Ghana: Who is at risk? *BMC Public Health* 13:288. 10.1186/1471-2458-13-288 23547846 PMC3620375

[B43] Stock J. H. Yogo M. (2002). *Testing for Weak Instruments in Linear IV Regression (NBER Technical Working Paper No. 284).* Cambridge, MA: National Bureau of Economic Research.

[B44] Tooley U. A. Bassett D. S. Mackey A. P. (2021). Environmental influences on the pace of brain development. *Nat. Rev. Neurosci.* 22 372–384. 10.1038/s41583-021-00457-5 33911229 PMC8081006

[B45] Vitellozzi S. Ferrone L. Otchere F. Poggi C. (2024). *Switching Roles: Gender Differences in Time Use and Mental Health in Ghana. AFD Research Papers.* Paris Cedex, France. 1–32.

[B46] von Grafenstein L. Kumar A. Kumar S. Vollmer S. (2023). Medium-run impacts of iron-fortified school lunch on anemia, cognition, and learning outcomes in India. *Oxford Bull. Econ. Statist.* 85 1262–1294. 10.1111/obes.12565

[B47] Wolicki S. B. Bitsko R. H. Cree R. A. Danielson M. L. Ko J. Y. Warner L. et al. (2021). Mental health of parents and primary caregivers by sex and associated child health indicators. *Advers. Resil. Sci.* 2 125–139. 10.1007/s42844-021-00037-7 36523952 PMC9749862

[B48] World Health Organization [WHO] (2022). *World Mental Health Report: Transforming Mental Health for All.* Geneva: World Health Organization.

[B49] Xerxa Y. Delaney S. W. Rescorla L. A. Hillegers M. H. J. White T. Verhulst F. C. et al. (2021). Association of poor family functioning from pregnancy onward with preadolescent behavior and subcortical brain development. *JAMA Psychiatry* 78 29–37. 10.1001/jamapsychiatry.2020.2862 32936235 PMC7495324

