# Supplementary material for: Household mental distress and child cognitive development in Ghana
Source: Front Psychol. 2026 Jul 29;17:1840678. doi: 10.3389/fpsyg.2026.1840678 (PMC13461410; doi:10.3389/fpsyg.2026.1840678)
Supplement: Supplementary file 1 [file Table_1.docx]

**Supplementary Appendix 1. Psychometric properties of the cognitive ability composite, including the principal component analysis, reliability statistics, and factor loadings.**

| Measure/Reliability Parameter | Item-Test Correlation | Item-Rest Correlation | Factor Loading/Value |
| --- | --- | --- | --- |
| Cognitive Indicators |  |  |  |
| Digit Span Forward | 0.86 | 0.672 | 0.761 |
| Digit Span Backward | 0.863 | 0.678 | 0.766 |
| Raven's Pattern Cognitive Assessment | 0.804 | 0.566 | 0.632 |
|  |  |  |  |
| Panel A: Scale Reliability Statistics |  |  |  |
| Cronbach's Alpha |  |  | 0.795 |
| McDonald's Omega |  |  | 0.765 |
| Average Inter-item correlation |  |  | 0.564 |
| Notes: Factor loadings are obtained from a one-factor model estimated with principal | | | |
| factor extraction. Cronbach’s alpha and McDonald’s omega assess internal consistency | | | |
| reliability. The average inter-item correlation is calculated across the three standardized | | | |
| cognitive assessments. |  |  |  |
